# Evolution of pathogen response genes associated with increased disease susceptibility during adaptation to an extreme drought in a *Brassica rapa* plant population

**DOI:** 10.1186/s12862-021-01789-7

**Published:** 2021-04-21

**Authors:** Niamh B. O’Hara, Steven J. Franks, Nolan C. Kane, Silas Tittes, Joshua S. Rest

**Affiliations:** 1 Jacobs Technion-Cornell Institute, Cornell Tech, New York, NY 10011 USA; 2 Department of Biological Sciences, Fordham University, Bronx, NY 10458 USA; 3 Department of Ecology and Evolution, The University of Colorado at Boulder, Boulder, CO 80309 USA; 4 Department of Ecology and Evolution, Stony Brook University, Stony Brook, NY 11794 USA

**Keywords:** Climate change, *Brassica rapa*, *Alternaria brassicae*, Drought, Resurrection approach, Necrotrophic fungal pathogen response

## Abstract

**Background:**

Pathogens are key components in natural and agricultural plant systems. There is evidence of evolutionary changes in disease susceptibility as a consequence of climate change, but we know little about the underlying genetic basis of this evolution. To address this, we took advantage of a historical seed collection of a *Brassica rapa* population, which we previously demonstrated evolved an increase in disease susceptibility to a necrotrophic fungal pathogen following a drought.

**Results:**

Previously, we combined a resurrection experiment with genome-wide sequencing of 124 pooled ancestral and descendant plants. Here, using these previously generated sequence data (Franks et al. in Mol Ecol 25(15):3622–3631, 2016), we show that well-characterized necrotrophic fungal pathogen response (NFPR) genes have evolved, as indicated by changes in allele frequency, between ancestors and descendants, with several of them identified as extreme F_ST_ outliers. The jasmonic acid (JA) signaling pathway in particular seems to underlie the evolution of disease susceptibility, in addition to its well characterized role in plastic disease response. We identify a list of 260 genes that are both NFPR genes and are differentially expressed in response to drought, based on publicly available data. We present evidence that five of these genes evolved between ancestors and descendants, suggesting that the drought acted as the evolutionary driver, and that the accompanying increase in disease susceptibility may have been a consequence of genetic pleiotropy.

**Conclusions:**

Our study provides evidence that for this population, standing variation in NFPR genes is affected by natural selection related to climate change. Our results reveal potentially important candidates that may underlie trait evolution in both crops and natural systems. Additionally, this trade-off between adaptation to biotic and abiotic stresses is an example of how climate change can have diverse and unexpected consequences.

**Supplementary Information:**

The online version contains supplementary material available at 10.1186/s12862-021-01789-7.

## Background

Pathogens exert strong selection on plants, often causing them to evolve increased resistance. Simultaneously, plant systems are exposed to a myriad of other selective pressures. Persistent climate change, such as long-term drought, may result in evolution of the hosts, pathogens, or both, leading to changes in virulence or susceptibility [1–5]. More broadly, exploration of how multiple, and at times opposing, selective pressures shape the genomes of plants is an important area of ongoing research.

The genetics of plant pathogen response is exceedingly complex and specific to whether the pathogen is necrotrophic or biotrophic [6, 7]. While response to biotrophs has been extensively studied and found to be generally mediated by a salicylic acid-dependent pathway, response to necrotrophic pathogens (the focus here) is less well understood and tends to have a more complex genetic architecture. Necrotrophic pathogens, which first kill tissue and then extract nutrients [8], stimulate a response pathway generally mediated by jasmonic acid (JA) and ethylene (ET) [9]. Such biotic stress response pathways have been shown to interact with abiotic stress response pathways. In particular, abscisic acid (ABA), which regulates growth and development in response to water availability, has been shown to suppress biotic stress response pathways, leaving plants more susceptible to pathogens [10]. ABA is thus an example of a regulatory system that may potentially be involved in adaptation to drought [11], and which may also result in pleiotropic evolution of pathogen susceptibility.

The most common approach used to characterize the genes responsible for pathogen response are expression studies in model plant-pathogen systems. However, low genetic variation, and distant phylogenetic relationships often limit the translation of such studies to crop breeding or characterization of evolving natural systems. Therefore, there is great promise in studying plant-pathogen genetics of natural systems. Some progress has been made in plant-pathogen systems with a simple genetic architecture including exploring genetic variation in resistance in natural populations for the *Linum-Melampsora* system [12], as well as exploration of resistance gene diversity within and between wild tomato populations [13, 14], and variation in specific resistance genes such as the RPW8 (Recognition of Powdery Mildew) [15]. But mostly, thus far, variation in resistance has been mapped at a spatial scale looking at local adaptation, rather than being followed over time [3, 12, 16]. The lack of focus on evolutionary trajectories limits our ability to predict evolutionary responses over short time scales. We addressed this limitation by taking advantage of evolutionary events, measuring changes in allele frequency, which allowed us to compare the genomes of ancestors and descendants to identify the genes involved in evolutionary changes in disease susceptibility in a natural system.

The system we used is the well-studied example of the adaptive evolution of earlier flowering in the annual herbaceous plant, *Brassica rapa* L. (syn. *campestris*) (Brassicaceae, field mustard) in response to a natural, extended drought [17–19]. Previous work used a resurrection approach [20, 21] in which *B. rapa* seed was collected in southern California in 1997, before an extended drought, and then again in 2004, post-drought. During the drought period, precipitation was substantially below-average, particularly later in spring, resulting in an abbreviated growing season [17]. Pre- and post-drought seeds were then grown under common conditions in the greenhouse and challenged with *Alternaria brassicae*. *A. brassicae*, which causes Alternaria blackspot disease, is a necrotrophic pathogenic sac fungus that causes damping off, leaf spots, defoliation and reduced seed yield [22]. We previously found that this population experienced a genetically-based evolutionary increase in Alternaria blackspot disease susceptibility [4], together with an adaptive shift to escape drought through earlier flowering [17]. While the shift to earlier flowering and drought escape was adaptive, the drivers for an increase in disease susceptibility remain unclear. Pathogen susceptibility may have evolved either due to direct selection, or indirectly as a result of selection on common genes [4, 23–25].

To explore the shift in disease susceptibility following a drought, we hypothesized that: (1) known necrotrophic fungal pathogen response genes (including on JA and ET pathways) evolved, and (2) pleiotropic genes, underlying pathogen and drought response evolved. To test these hypotheses we used genome sequences, generated in our previous work [26], of an ancestral (pre-drought) and descendant (post-drought) *B. rapa* natural population, and explored whether necrotrophic fungal pathogen response (NFPR) genes evolved. We assessed signatures of evolution in two complementary ways. First, we conducted genome-wide outlier F_ST_ analysis to determine if allele frequencies of previously characterized NFPR genes were differentiated between ancestors and descendants, indicating rapid contemporary evolution at these loci. Second, we used site frequency spectrum analysis (Tajima’s D) to look for historical signatures of selective sweeps or other strong or recurrent selective patterns at NFPR genes. Finally, we identified a list of genes that were both NFPR genes and were differentially expressed in response to drought, based on publicly available data. We used this list to consider the potential role of pleiotropic evolution of both NFPR and drought response.

## Results

Previously we identified 5,812,602 SNPs (out of 235,128,010 sites), including SNPs in 35,202 genes using a low-coverage approach (~ 25× per pooled library). The general results of our sequencing and our observation of global and flowering time related allele frequency shifts have been published elsewhere [26]. In that study, we found an expected heterozygosity of 0.5–0.6 across the pools, with no major reductions in genetic diversity or heterozygosity, indicating that the drought likely did not cause a genetic bottleneck. Also, we observed an evolutionary shift in disease susceptibility for this population following the drought event [4]. In the present study, we re-analyzed our genomics data for candidate NFPR genes in order to explore the genetic basis of this evolutionary shift in disease susceptibility. We found evidence of evolution in NFPR genes, demonstrated by a change in allele frequencies at these pathogen response loci between ancestor and descendant populations, and that the JA signaling pathway seemed to underlie the evolution of disease susceptibility. We found that a number of these NFPR genes were also responsive to drought, suggesting the potential role of pleiotropic evolution.

### Evolution of NFPR and NFPR/drought response genes

**Fig. 1:**
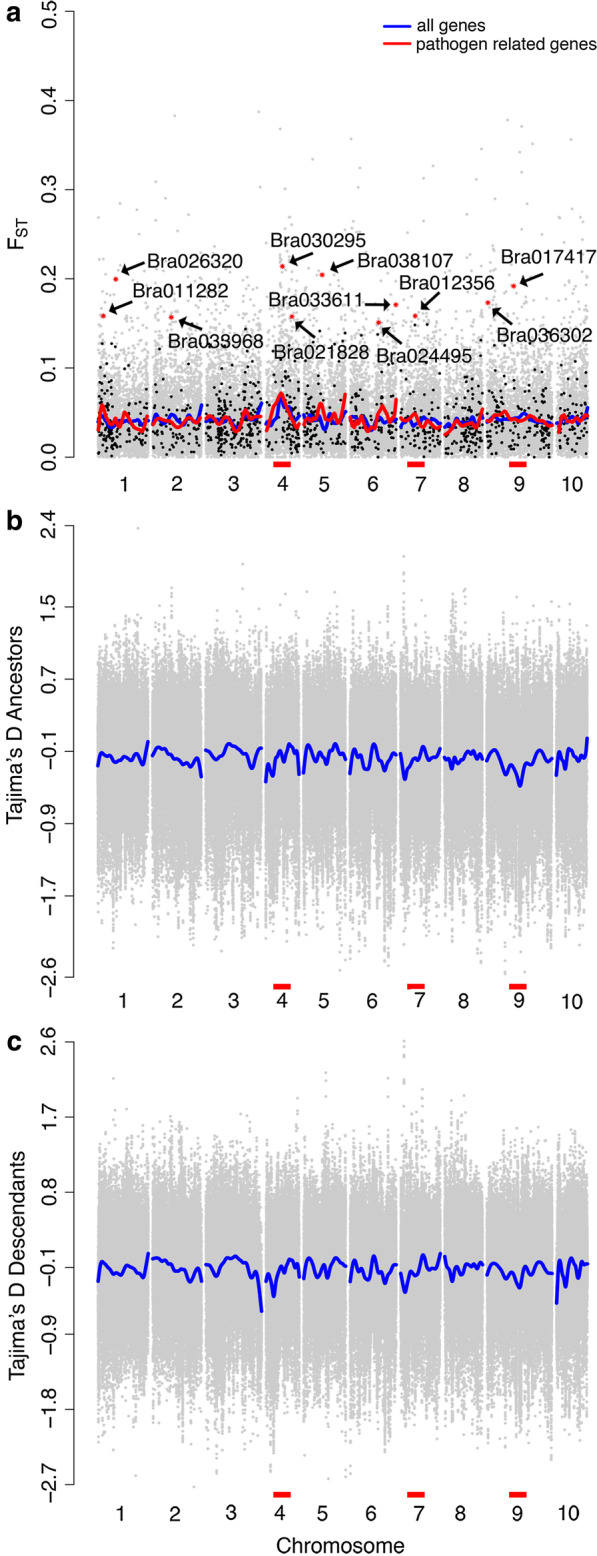
Differentiation at multiple loci across the genome in a *Brassica rapa* population after seven years of drought, as well as historical measures of selection. **a** F_ST,_ an estimate of allelic differentiation between the 1997 and 2004 populations, was calculated using 100 kb sliding windows and then averaged for each gene (dots shown). Genes reported in the literature as involved in response to necrotrophic fungal infection (*A. brassicicola*) are shown in black. All other genes are shown in grey. For necrotrophic fungal pathogen response genes, significantly differentiated genes are labelled with red points and their gene ID. A LOESS trend line (span = 0.03) is shown for all genes (blue) and just for necrotrophic fungal pathogen response genes (red). Tajima’s D, a statistic estimating the effect of non-random processes from the site frequency spectrum, shows historical selection across the genome for the **b** ancestral population and **c** descendant populations. Analysis was conducted using a 100 kb sliding window (dots shown are windows; LOESS trend line in blue). Red bars highlight regions containing necrotrophic pathogen related genes with a significant high F_ST_ that are also in an area with reduced Tajima’s D (visual inspection). Regions with reduced Tajima’s D may have been subject to a historical selective sweep

To explore our first hypothesis, that pathogen response genes evolved, we used an a priori approach, and found that 11 genes from of our database of 1182 candidate NFPR genes were significantly differentiated between ancestors and descendants (Fig. 1), with an F_ST_ above background compared to all the genes, as determined by outlier F_ST_ analysis (q < 0.05). These genes were found throughout the genome on the majority of *B. rapa*’s 10 chromosomes, including chromosomes 1, 2, 4, 5, 6, 7, and 9. In contrast, in the entire genome, we saw 434 out of 35,202 genes evolved [26]. The NFPR genes that evolved were not over-represented among all genes that showed significant evolution (Chi-square = 0.846, p = 0.358). The mean F_ST_ for all NFPR genes was not significantly elevated above background (ANOVA: F_1,35199_ = 0.008, p = 0.929). In a Manhattan plot comparing F_ST_ of NFPR to all other genes across the genome, the NFPR and all genes’ trend lines tracked one another (Fig. 1), in fact 95% confidence intervals (not shown) overlapped across the whole genome. We report the 20 most differentiated NFPR genes (Table 1; see Additional file 1 for top 50). This includes the 11 genes that were significant, plus additional genes that could potentially have been involved in the shift in susceptibility despite not being statistically significant after false discovery correction.

**Table 1:** The 20 most highly differentiated (high F_ST_) necrotrophic fungal pathogen response genes

*A. thaliana* ID	*B. rapa* ID	F_ST_	q value	Gene name	Notes	Description
**AT2G21520**	**Bra030295**	**0.21**	**1.75 × 10**^**–5**^		**1**	**Sec14p-like phosphatidylinositol transfer family**
**AT3G15210**	Bra038107*	**0.20**	**7.30 × 10**^**–5**^	**ERF4, RAP2.5**	**1,2**	**Ethylene responsive element binding factor 4**
**AT5G54160**	Bra026320*	**0.20**	**1.45 × 10–**^**4**^	**OMT1**	**1**	***O-methyltransferase 1***
**AT2G02390**	**Bra017417**	**0.19**	**4.16 × 10**^**–4**^	**GST18,GSTZ1**	**1**	**Glutathione S-transferase zeta 1**
**AT4G01960**	**Bra036302**	**0.17**	**4.48 × 10**^**–3**^		**1**	
**AT4G39270**	Bra033611*	**0.17**	**5.63 × 10**^**–3**^		**1**	**Leucine-rich repeat protein kinase family**
**AT4G31550**	Bra011282*	**0.16**	**0.02**	**WRKY11**	**1**	**WRKY DNA-binding protein 11**
**AT1G23040**	Bra012356*	**0.16**	**0.02**		**1**	**Hydroxyproline-rich glycoprotein family protein**
**AT2G33120**	**Bra021828**	**0.16**	**0.02**	**SAR1,VAMP722**	**1**	**Synaptobrevin-related protein 1**
**AT1G67980**	**Bra033968**	**0.16**	**0.03**	**CCOAMT**	**1,2**	**Caffeoyl-CoA 3-O-methyltransferase**
**AT2G17720**	**Bra024495**	**0.15**	**0.05**		**1**	**2-Oxoglutarate (2OG) and Fe(II)-dependent oxygenase superfamily protein**
AT1G45145	Bra036335	0.15	0.06	ATH5,LIV1,TRX5	1,2	Thioredoxin H-type 5
AT1G74020	Bra003820	0.15	0.07	SS2	1,2	Strictosidine synthase 2
AT1G23730	Bra012384	0.15	0.07	BCA3	1	Beta carbonic anhydrase 3
AT4G12720	Bra012746	0.14	0.11	GFG1,NUDT7	1	MutT/nudix family protein
AT2G02930	Bra018543	0.14	0.14	GST16,GSTF3	1	Glutathione *S*-transferase F3
AT1G10140	Bra031702	0.14	0.16		1,2	Uncharacterised conserved protein UCP031279
AT3G02520	Bra040592	0.14	0.17	GF14 NU,GRF7	1	General regulatory factor 7
AT5G65750	Bra024417	0.14	0.19		1	2-Oxoglutarate dehydrogenase, E1 component
AT1G16520	Bra026043	0.14	0.22		1	

Significantly differentiated genes (based on q value) are bolded. Asterisks show significantly differentiated genes that are involved in both drought and pathogen stress response [27, 28], as determined by expression analysis (1), or where genes have been shown to be *COI1* dependent (2). Descriptions were retrieved from http://brassicadb.org/brad/

**Fig. 2:**
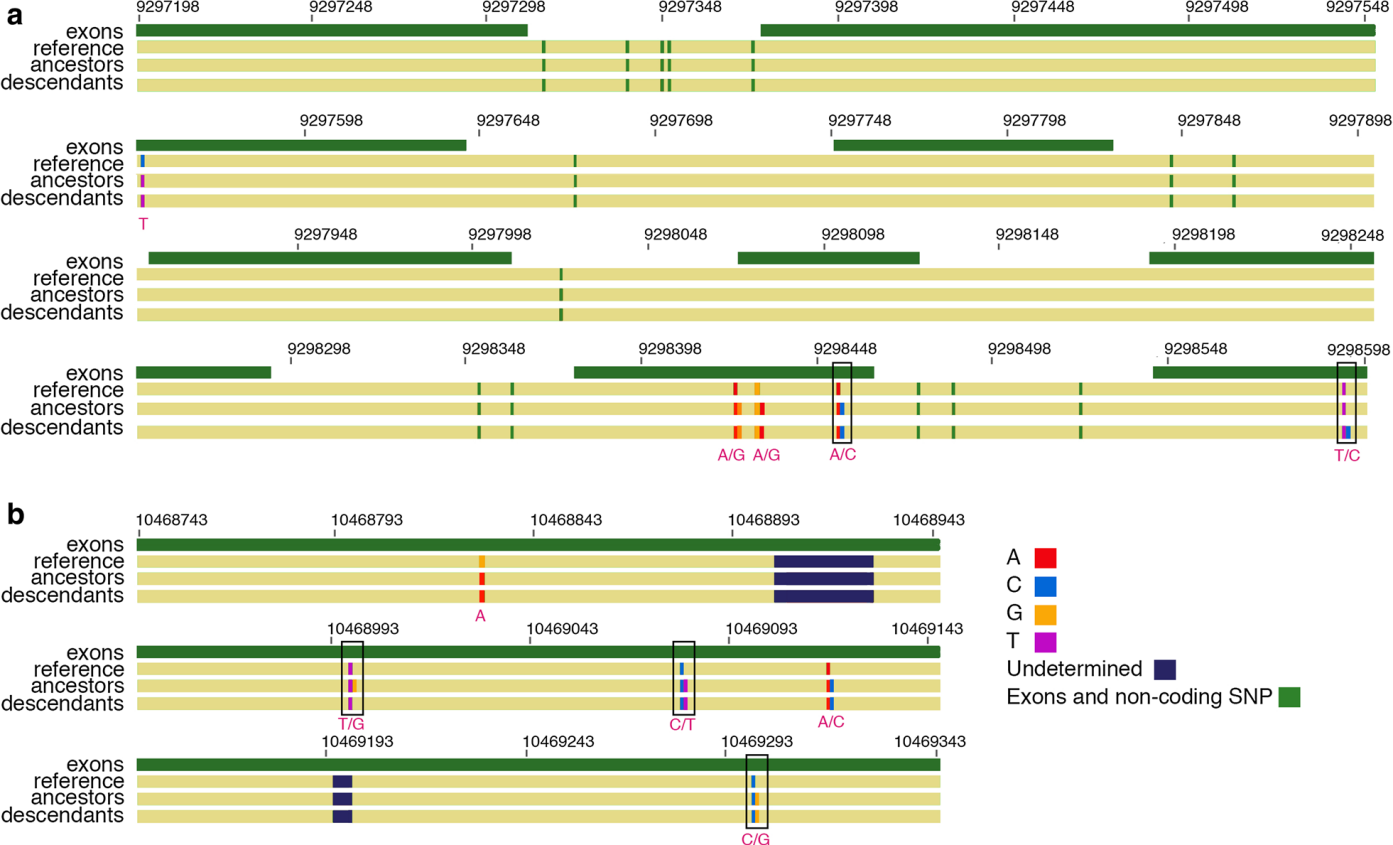
Mapping of SNPs and designation of synonymous and non-synonymous (outlined in black box) SNPs in our two top candidate necrotrophic fungal pathogen response genes in *Brassica rapa* that evolved over seven years of drought. Shown are alignments between *B. rapa* reference V. 1.18, ancestral *B. rapa* and descendant *B. rapa* for our 2 top candidate genes in top and bottom panels, with exons and non-coding SNPs shown in green. Nucleotide identities in coding regions are indicated below SNP sites and identified by the following colors: red for A, bright blue for C, orange for G, and purple for T. Heterozygous sites are indicated by the presence of different adjacent colors and indicated by including both nucleotides below heterozygous sites (e.g. A/T). Allele frequencies are not indicated. Undetermined regions are shown in dark blue. **a** Bra030295 had 19 SNPs, 5 of which are in exons, and 2 of which are non-synonymous (black boxes), indicating candidate functional polymorphisms. **b** Bra038107 had 5 segregating sites, all in the single exon, with 3 non-synonymous SNPs (black boxes), indicating candidate functional polymorphisms

To explore our second hypothesis, that genes involved in both NFPR and drought response evolved, we also used an a priori approach and found that 5 (2.0%) of the genes from our joint list of 260 genes that responded to both conditions were significantly differentiated between ancestors and descendants, with an F_ST_ above background compared to all genes (q < 0.05) (Table 1). These five genes did not constitute an over-representation among all genes that showed significant evolution (Chi-square = 0.974, p = 0.324). We show the overlap in NFPR, drought response, and genes that evolved (demonstrated by significant F_ST_ values) in a Venn diagram (Fig. 2). This list of genes that evolved, and that are associated with both NFPR and drought response, are candidates genes for pleiotropic evolution (i.e. where genetic change at a single locus affected both phenotypes).

### Historical selection

To determine if the genes that were highly differentiated between 1997 and 2004 were also under selection historically, we calculated the Tajima’s D value for each gene. We found that none of the highly differentiated genes also had a significantly reduced Tajima's D value. There was no difference in Tajima’s D between significantly differentiated (F_ST_) NFPR genes and the rest of the NFPR genes (ANOVA: ancestors F_1,1180_ = 2.398, p = 0.122; descendants F_1,1180_ = 0.551, p = 0.458). This was consistent with our genome-wide result, where we found little overlap between genes with high F_ST_ and negative Tajima’s D [26]. These findings were not surprising because these estimates detect selection acting at different time scales. Visual inspection of Tajima's D values over the genome suggested that three of our significant (F_ST_) NFPR genes were in regions of reduced Tajima’s D (red bars in Fig. 1), which could indicate they were linked to regions that may have experienced historical selective sweeps, although the values of Tajima’s D for the genes of interest were themselves not significantly reduced.

**Fig. 3:**
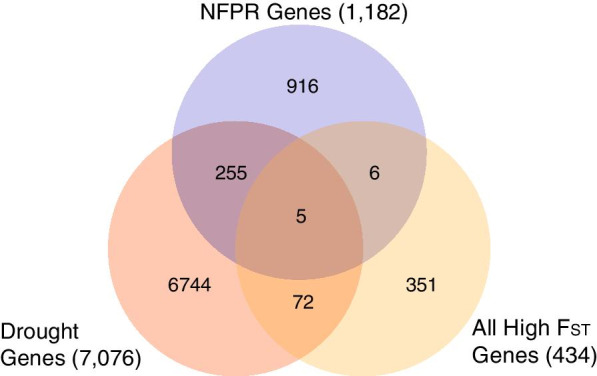
Venn diagram showing gene databases used in this study for annotation including necrotrophic fungal pathogen response genes (NFPR), drought response genes (Drought), genes which evolved genome-wide (Sig Genes), and pleiotropic genes (overlap between NFPR and drought response) out of a total 35,202 genes

We next explored the nature of segregating sites in two NFPR candidate genes with the highest F_ST_ values, Bra030295 and Bra038107. We explored whether polymorphism in these genes would result in changes in the amino acid sequence, and therefore potentially be a direct subject of selection. We made alignments of the ancestral, descendant, and reference sequences for these genes (Fig. 3). We determined whether sites were in exons or introns and, if in exons, whether they were synonymous or nonsynonymous. These genes were also in regions that had reduced Tajima’s D, by visual inspection, or have important functions (see “Discussion”). We found that the two genes together had 10 SNPs in exons, and 5 of these exonic SNPs were nonsynonymous. Bra030295 had two and Bra038107 had three nonsynonymous sites that experienced an allele shift between ancestors to descendants.

### Jasmonic acid and the evolution of disease susceptibility

Since the JA pathway is such an important mediator of necrotrophic fungal pathogen response, we specifically explored whether JA (and downstream gene *COI1*) may be involved in the evolution of disease susceptibility. We found that 17 (34%) out of our top 50 most NFPR differentiated genes were *COI1* and/or JA dependent (Additional file 1). This is similar to the ratio 441 (37%) out of 1182 of COI1/JA genes which are differentially expressed in response to pathogens in van Wees et al. [27] and Mukherjee et al. [28] pathogen studies; we found that these ratios are statistically equivalent (Chi-square = 0.225, p = 0.635), despite the very different approach of analysis using resurrection/sequencing instead of gene expression.

### Gene ontology analysis

We used gene ontology analysis to determine if pathogen response functional categories are overrepresented globally among all genes when ranked by their level of evolutionary differentiation between ancestors and descendants (F_ST_). We found that pathogen response categories were not significantly enriched and were not highly ranked out of the 3136 categories. The top pathogen response category was “plant-type hypersensitive response” (GO:0009626), ranked 54th (p = 0.014, Benjamini–Hochberg FDR corrected p = 0.835), followed closely by “host programmed cell death induced by a symbiont” (GO:0034050), ranked 56th (p = 0.015, Benjamini–Hochberg FDR corrected p = 0.822).

## Discussion

In previous work, we found that a natural *B. rapa* plant population in southern California evolved earlier flowering [17] and increased fungal disease susceptibility [4] following a 7-year natural drought. A shift in susceptibility for this plant pathogen system could have widespread implications because this fungus is an important agricultural problem for *B. rapa* crops [29, 30]. Also, we have found widespread infection (22.2% of plants observed to be infected) in all 6 of our *B. rapa* field populations on the west coast of the USA [31]. In the current study, we compared the genomics of ancestral and descendant plants from one population to elucidate the underlying genetic and molecular basis of this shift in disease susceptibility. This is the first study, to our knowledge, to examine the genetic basis of evolution of susceptibility to pathogens in a natural plant population following drought [32–35]. There are many challenges to studying evolution in natural systems. Using a resurrection genomics approach we were able to both directly measure the disease susceptibility of ancestors and descendants in common conditions, and directly assess evolution by measuring changes in allele frequencies between these ancestral and descendant populations [26].

### Evolution of NFPR genes

Using an outlier F_ST_ analysis approach, we found evidence for our first hypothesis, that a number of NFPR genes evolved over the course of just 7 years of drought. This is consistent with selection acting on a trait that is controlled by multiple loci, such as defense response to a necrotrophic fungus. This is expected to be more complex than response to a biotrophic pathogen, which in many cases is controlled by a single gene [6, 36]. We did not see significant evolution across the wider NFPR system compared to the rest of the genome. It is possible, given the genetically complex nature of NFPR, that specific causative genes could evolve, underlying a shift in susceptibility, without evolution across the wider system, especially given the short time frame between ancestors and descendants studied. In addition, we found that a substantial proportion of these NFPR genes are JA/*COI1* dependent. Studies in the closely related *A. thaliana*–*A. brassicicola* model plant pathogen system have identified *COI1*-dependent genes, part of the JA signalling pathway, as playing a central role in defense against necrotrophic fungal pathogens, suggesting that JA signalling mediates the defense response. JA activity is dependent on the downstream *COI1* gene*,* which encodes an F-box protein involved in proteolysis [37]. JA signalling might play an important role in the evolution of susceptibility in addition to its known role in a plastic response to necrotrophic fungal pathogens.

There are limitations to using an outlier F_ST_ analysis to characterize the ultimate molecular or developmental mechanisms by which a population phenotypically evolves (e.g. driving mechanism of evolution). Additionally, our pooling sample size may limit us to identifying alleles with larger changes in allele frequency. However, our outlier F_ST_ approach, together with a q-value threshold correction, is effective at characterizing loci that showed significant shifts, and thereby can identify putative candidate genes underlying genetically-based phenotypic shifts observed. This approach has also been successfully utilized in other studies in these populations [26].

### Potential signatures of pleiotropic evolution

In previous work, we found adaptive evolution in response to drought (through earlier flowering) also resulted in the evolution of pathogen susceptibility in this population [4]. In exploration of this associated response, in the current study, we also found evidence for our second hypothesis: that a number of genes involved in both drought and pathogen response evolved, suggesting a signature of pleiotropic evolution. In the current study, we considered genes to be candidates for pleiotropic evolution for drought and necrotrophic fungal pathogens if they had been found to be differentially expressed in response to independent drought stress and inoculation with a necrotrophic fungal pathogen, and if in our study they evolved between ancestors and descendants. A subset of five of these genes evolved in our study. Allelic variation in any of these five genes may have simultaneously affected both drought response and NFPR response, i.e. caused pleiotropic phenotypic changes. For this population we found clear evidence that JA pathway genes, which affect both pathogen response, flowering time, and stress resistance [38–40], evolved, suggesting evidence for evolution in pleiotropic genes in this system. These findings agree with other studies which have found that pleiotropy plays a role in shaping multiple ecological traits for plant populations displaying adaptation to local water availability [41]. Furthermore, it is possible that antagonistic pleiotropy was playing a role in the evolution observed [42]. Under this scenario, pleiotropic genes underlying both drought and pathogen response could have evolved under positive selection due to drought, despite negative selection by pathogens, if the negative selection was not strong enough to overcome the positive selection, or if it was not acting in the contemporary environment [4]. Indeed, the increased susceptibility we observed in this population was accompanied by an evolutionary shift to thinner leaves (increased specific leave area) [4], which could indicate a trade-off between drought escape and disease susceptibility in agreement with the growth and defense trade-off [43, 44].

### Historical selection

In addition to examining evolution following the recent drought, we tested whether NFPR genes were under historical directional selection by looking for genes with a low Tajima's D (< − 2). Loci under balancing selection would not have a low Tajima's D. Genome-wide, we found no association between F_ST_ and Tajima’s D, and there were no genes that were outliers for both high F_ST_ (> 0.2) and low Tajima’s D (< − 2). This finding concurs with other prior work in this system [26]. This indicates that evolution in response to the drought affected different genes in this population than have historically been the target of directional selection.

### Top candidate genes underlying evolutionary shift in susceptibility

The most highly differentiated gene in this study was Sec14p-like phosphatidylinositol transfer family protein (Bra030295) (Fig. 3a; F_ST_ = 0.21; q = 1.75 × 10^–5^) which has been found to be differentially expressed in *Arabidopsis* in response to infection with *A. brassicicola *[27] and cabbage leaf curl virus [45]. Little is known about the specific function of this gene, although it is thought to play a role in the movement of substances across cell membranes and has been found to be expressed widely in 23 plant structures [46]. The shift in allele frequency at two non-synonymous segregating sites in this gene indicate a potential functional effect, with one of these polymorphisms being shared between ancestors and descendants (potentially indicating a previous shift due to the recurrent droughts in this region), and the other polymorphism found only in the descendant population, indicating a better functional candidate in response to this drought event.

Several of the NFPR genes with the highest level of differentiation between ancestors and descendants demonstrate the pleiotropic nature of the genes subject to evolution in response to the drought in this population. We found that the second most highly differentiated NFPR gene was the *COI1* dependent gene ERF4 (Bra038107) (F_ST_ = 0.20; q = 7.30 × 10^–5^). In addition to a demonstrated role in the antagonistic regulation of JA and ET, this gene is also induced by ABA [47, 48]. This is interesting because this gene, along with other genes mediated by ABA that we found to have high F_ST_, including Bra012746 and Bra033968, and Bra011282, have pleiotropic roles in both abiotic and biotic stress response [49–51]. Studies have shown that ERF4 is induced by ABA, and ERF4 suppresses expression of PDF1.2 [52], a defense effector that is elicited by JA signaling [6]. This gene showed a shift in allele frequency at three non-synonymous sites, and evolution at these sites could have a functional effect (Fig. 3b). Two of these polymorphisms were shared between ancestors and descendants (potentially indicating a previous shift due to the recurrent droughts in this region), and the third polymorphism was found only in the descendant population, indicating a better functional candidate in response to this drought event.

Caffeoyl-CoA 3-*O*-methyltransferase (Bra033968) is an NFPR gene that is *COI1* dependent [27] and also had a significant F_ST_ value. It is a lignin biosynthesis gene, which is pleiotropic, playing a role in both drought and pathogen response. Lignification is a primary defense mechanism against pathogens [53], which works primarily through leaf structure [54]. It has also been shown to be suppressed in Arabidopsis by ABA application, along with other pathogen response genes, resulting in a decrease in lignin accumulation and an increase in disease susceptibility to *Pseudomonas syringae pathovar (pv.) tomato *[55]. This gene is also interesting because lignin plays a key role in leaf structure, and we found evidence that leaf structure played a role in the increase in disease susceptibility seen [4], with earlier flowering plants having thinner leaves that displayed greater disease severity.

## Limitations of this study

This study was limited due to it being an observational study focused on one natural population that underwent an evolutionary shift in response to an extended drought. Alternative explanations to pleiotropy include that drought response and pathogen susceptibility were acted on separately, or that if there is a large cost of disease resistance and these pathogens become less common during a drought, then selection could favor reduced resistance due to these costs. The current study design does not allow us to differentiate between these alternative explanations. Experimental evolution studies with additional populations, including a non-drought control population will be important to address some of these findings further. Additionally, epigenetics, especially longer-term stable epigenetics could have played a role in the shift observed, and was not tested in the present study. Such epigenetic effects may act in addition to or in concert with the observed genetic changes, and would not replace or negate the contribution of genetic change. Although seeds from prior generations emerging from the seedbank could have influenced our findings, this effect is likely minimal and would only have caused our analyses to be more conservative because less evolution would be observed than actually occurred [17]. Finally, in addition to pleiotropy, correlated change in these two phenotypes could have been caused by drift, linkage, or by simultaneous change in selective pressure on both flowering time and pathogen load. While we cannot eliminate these from consideration, they likely played a minor role in the observed patterns due to data presented in a previous study showing an adaptive evolution in response to drought, lack of evidence of a bottleneck, and the short 7-year time frame over which the evolution occurred, too short for drift alone to explain the extensive allelic differentiation observed [26].

## Conclusions

The results from our resurrection genomics study demonstrate that many well-characterized candidate NFPR genes evolved, with a change in allele frequency, in just 7 years of drought. We conclude that *COI1* and the JA pathway are likely to be involved in the evolution of disease susceptibility in this population. These genes are thus candidates for future investigation of variation in disease susceptibility in other crop and wild populations of this and related species. Several implicated genes are potentially involved in both defense and drought resistance, suggesting that antagonistic pleiotropy potentially caused the observed evolutionary increase in disease susceptibility following a natural drought. That this previously unexplained increase in disease susceptibility is a result of adaptation to drought represents a surprising response of a plant population facing changes in local climate, and demonstrates that tradeoffs among fitness components can explain seemingly maladaptive responses.

## Methods

## Plant samples

In prior work, a large number of seeds (> 10,000) were bulk collected from a natural feral *B. rapa* population (Arboretum) in Southern California near the University of California Irvine campus. Seeds were collected in 1997, before an extended natural drought, and then again in 2004, post-drought. Before generating data, plants were previously grown from seed and cross-pollinated to control for maternal and storage effects [17].

### Sample preparation and shotgun sequencing

The pooled sequence data used in this study was generated in our previous work and described in more detail there in Franks et al. [26]. To summarize, previously, 500 ancestral and descendant plants (total N = 1000) were grown up from F2 seeds, tissue was collected, genomic DNA was extracted, quality-checked, and quantified by real-time PCR. Samples were pooled in equimolar amounts, in duplicate, within year (ancestral N = 50, descendant N = 74), and sequenced on a HiSeq 2000 (Illumina). Note that Franks et al. [26] also included a second population (Back Bay) not studied here, and for which we do not have disease susceptibility data. We have focused on the Arboretum population for disease susceptibility studies [4] due to the greater evolutionary shifts in multiple traits experienced by that population [17].

### Bioinformatic analysis and validation

We previously used computational methods described in Franks et al. [26] to trim, clean and prepare our reads for analysis including aligning sequence reads to the *B. rapa* genome version 1.18 (http://brassicadb.org/brad/) [56], calling sequence variants, and calculating population genetic estimates including divergence (F_ST_) [57, 58] and Tajima's D [59] across 100-kb windows. In this previous work, steps were taken to improve confidence in calling SNPs and population genetic estimates, including using a minimum of 8× coverage across each window, and removal of singletons SNPs. Pooling design, including choosing number of samples to pool to generate sufficient coverage, was informed by modelling and empirical studies that show precision and accuracy of population parameters (e.g. allele frequency) to be maximized when a low coverage (i.e. 0.5×–1×) population-level (> 20 individuals) approach is used [60]. Population genetic estimates were compared between pooling replicates and found to be highly correlated, so these technical replicates were merged for all reported analyses. SNPs for these pooled whole-genome sequences were also validated in our previous study using Kompetitive Allele-Specific PCR genotyping (KASP) on SNPs in 10 genes for 116 individual plants, with allele frequencies obtained by KAPS-genotyping on individuals showing high correlation with pooled Illumina sequencing [26].

In our previous work, statistical significance of genes, results which are used here, were determined for the F_ST_ values of genes by comparing the experimental F_ST_ distribution to a null distribution created by resampling and regressing the experimental distribution on a null distribution to calculate p values for residuals. After multiple test correction, genes were considered significant if they had a q value of less than 0.05 [61]. We also report the top 50 most differentiated NFPR genes, including those with a q value of > 0.05.

### Plant-pathogen system

We considered previously published observations from the compatible plant-pathogen system *Brassica rapa*–*Alternara brassicae* in this study, as described in detail in our previous work [4]. The molecular mechanisms of pathogen response in the *Brassica rapa*–*Alternaria brassicae* system is not well understood [62], however, extensive research has been conducted on the closely related plant pathogen system *Arabidopsis thaliana*–*Alternaria brassicicola*, which is a model system used for studying diseases caused by necrotrophs [6, 27, 28]. In the current study, we took advantage of this extensive model system work and used an a priori approach [63] to characterize the evolution of a set of well-researched candidate disease-related genes in our resurrected ancestral and descendent natural populations, as described in more detail in the next section. Studies in the *A. thaliana–A. brassicicola* system have identified a large number (~ 1100) of candidate NFPR genes [6, 27, 28]. Also, these studies found that JA is required for resistance, indicated by susceptibility in mutants with non-functional copies of genes involved in the JA pathway, including a mutation in the downstream *COI1* gene (see Glazebrook [6] for further details). Additionally, a recent *B. rapa* expression study was used to identify candidate drought response genes [64].

### Functional analysis

In the current study, for our a priori analysis approach, a database of 1182 well-characterized candidate NFPR genes (Additional file 2) was assembled from two previous *A. thaliana*–*A. brassicicola* studies by Mukherjee et al. [28] and van Wees et al. [27]; results from these studies were used for functional annotations in our study. These studies were chosen in order to span the range of compatibility that exists between *B. rapa*–*A. brassicae *[36], including genes shown to be involved in both compatible interactions [28] and incompatible interactions [27]. Briefly, to identify candidate NFPR genes, van Wees et al. [27] used mutants with non-functional copies of genes involved in JA and SA signaling followed by inoculation and expression profiling, and Mukherjee et al. [28] used suppression subtractive hybridization (SSH) to identify differential expression between compatible and incompatible plant lines challenged with the pathogen. For both studies, which measured differential expression across the whole transcriptome, NFPR candidate genes were determined as genes with differential expression between plants that were inoculated with the fungal pathogen and those that received a mock inoculation. We used this database for functional annotations in our study.

We next considered 7076 genes candidate drought response genes (Additional file 3) which were differentially expressed in Guo et al. [64] study in response to a polyethylene glycol (PEG) treatment, as compared to controls (no PEG), and that had a > twofold change at 12 h post-treatment. We then identified genes differentially expressed in common among both this drought study and the NFPR studies (above). In total, we found 260 genes were significantly differentially expressed in both types of conditions.

Lastly, we conducted a functional analysis by analyzing gene ontology (GO) using ErmineJ [65], with annotations from *Arabidopsis thaliana*. To analyze our F_ST_ distribution, which was non-normal, we used ranked gene scores in a receiver operator characteristic (ROC) analysis and default ErmineJ settings. p values were corrected using a Bejamini–Hochberg false discover rate (FDR) correction.

## Supplementary Information

**Additional file 1.** Table of top 50 most differentiated necrotrophic fungal pathogen response genes including F_ST_ value, gene description, and notes on pathogen response pathway from previous studies.

**Additional file 2.** Table of necrotrophic fungal pathogen response genes from Mukherjee et al. [28] and van Wees et al. [27] used in our a priori analysis.

**Additional file 3.** Table of necrotrophic fungal pathogen response genes from Guo et al. [64] used in our a priori analysis. These genes were differentially expressed in the Guo et al. [64] study at 12 h in response to a polyethylene glycol (PEG) treatment, as compared to controls, and had a > twofold change.

## Data Availability

The dataset supporting the conclusions of this article are included within the article (and its additional files).

## Abbreviations

COI1: Coronatine-insensitive 1
JA: Jasmonic acid
ET: Ethylene
ABA: Abscisic acid
NFPR: Necrotrophic fungal pathogen response
SNP: Single nucleotide polymorphism
SSH: Suppression subtractive hybridization
PEG treatment: Polyethylene glycol treatment
GO Analysis: Gene ontology analysis
FDR: False discover rate
